# Fc-Receptor Targeted Therapies for the Treatment of *Myasthenia gravis*

**DOI:** 10.3390/ijms22115755

**Published:** 2021-05-28

**Authors:** Christian W. Keller, Marc Pawlitzki, Heinz Wiendl, Jan D. Lünemann

**Affiliations:** Department of Neurology with Institute of Translational Neurology, University Hospital Münster, 48149 Münster, Germany; kellerch@ukmuenster.de (C.W.K.); Marc.Pawlitzki@ukmuenster.de (M.P.); heinz.wiendl@ukmuenster.de (H.W.)

**Keywords:** *myasthenia gravis*, antibody, IgG, Fc, Fc receptor, immunotherapy, personalized medicine

## Abstract

*Myasthenia gravis* (MG) is an autoimmune disease in which immunoglobulin G (IgG) antibodies (Abs) bind to acetylcholine receptors (AChR) or to functionally related molecules in the postsynaptic membrane at the neuromuscular junction. IgG crystallizable fragment (Fc)-mediated effector functions, such as antibody-dependent complement deposition, contribute to disease development and progression. Despite progress in understanding Ab-mediated disease mechanisms, immunotherapy of MG remained rather unspecific with corticosteroids and maintenance with immunosuppressants as first choice drugs for most patients. More specific therapeutic IgG Fc-based platforms that reduce serum half-life or effector functions of pathogenic MG-related Abs are currently being developed, tested in clinical trials or have recently been successfully translated into the clinic. In this review, we illustrate mechanisms of action and clinical efficacies of emerging Fc-mediated therapeutics such as neonatal Fc receptor (FcRn)-targeting agents. Furthermore, we evaluate prospects of therapies targeting classical Fc receptors that have shown promising therapeutic efficacy in other antibody-mediated conditions. Increased availability of Fc- and Fc receptor-targeting biologics might foster the development of personalized immunotherapies with the potential to induce sustained disease remission in patients with MG.

## 1. Introduction

*Myasthenia gravis* (MG) is the prototypical antibody-mediated autoimmune disease. Pathogenic immunoglobulin G (IgG) antibodies (Abs) bind to acetylcholine receptors (AChR) or to functionally related molecules in the postsynaptic membrane at the neuromuscular junction and induce localized or generalized weakness of skeletal muscles. With an annual incidence of 10 cases per 1 million persons and a prevalence of 250 cases per 1 million, MG and its various subgroups (see also Section 4) are the major diseases that affect the neuromuscular junction [1]. Although the diagnosis is straightforward in most patients with typical symptoms and a positive Ab test, the phenotype of the disease and its clinical course including the response to immunotherapy is remarkably heterogeneous. Variants of MG are defined on the basis of disease phenotype including severity, age of disease onset, response to first-line therapies and these subgroups influence therapeutic decisions and prognosis (see also Section 4).

Unlike other molecules in the immune system, Abs consist of two distinct functional domains: (1) the antigen-binding domain (Fab) conferring antigen-specificity and binding and (2) the constant fragment crystallizable (Fc) domain providing instructions to the immune system. Pathogenic functions of AChR-specific Abs mediated by their Fab domain include blockade of ACh binding to the AChR resulting in inhibition of ACh-dependent signaling at the neuromuscular junction and internalization of the AChR following autoantibody-mediated crosslinking [2]. Fc domain-mediated effector functions of AChR Abs, which predominantly belong to complement-fixing IgG1 and IgG3 subclasses, include activation of complement at the postsynaptic membrane resulting in AChR loss and destruction of its characteristic architecture, which is necessary for efficient signal transduction. The remarkable clinical efficacy of pharmacological complement inhibition in MG highlights the importance of IgG-Fc mediated complement activation in MG pathology [3,4,5]. Cellular infiltrates (mostly macrophages and T cells) are present in MG skeletal muscle biopsies [3]. These are rarely topographically related to the neuromuscular junction [3] but are believed to contribute to MG pathology by production of soluble immune factors both locally within the muscle environment and systemically, leading to increased serum levels of proinflammatory cytokines [5,6]. Such cellular immune functions are regulated by signaling through Fcγ receptors (FcγR) expressed by many leukocyte subsets [7].

Therapeutic platforms targeting Fc-mediated functions through classical FcγRs and the non-classical major histocompatibility complex (MHC) class I-related neonatal FcR (FcRn), which regulates IgG serum half-life, are currently being developed, tested in clinical trials or have been successfully translated into the clinic for the benefit of patients with MG.

Targeted immunotherapy seems to be the most promising therapeutic approach in MG because it can effectively overcome the limitations of current nonspecific immunotherapies and has the potential to induce remission. Here, we illustrate the rationale and potential for Fc- and FcR-targeting biologics to treat MG.

## 2. MG Pathophysiology

MG is a complex immune-mediated disease characterized by circulating Abs that target molecules in the postsynaptic membrane at the neuromuscular junction. Dysregulated activation of T helper (Th)17 and follicular Th cells as well as impaired regulatory T cell (Tregs) function promote MG pathology [8]. Predisposing genetic factors (such as associations with HLADRB1*1501, HLADQ5 and CTLA4 polymorphisms) influence onset and course of disease while the role of triggering environmental cues remains controversial [1].

Approximately 85% of MG patients have detectable Abs against the muscle nicotinic AChR. This major MG subgroup (AChR MG) encompasses early-onset (EOMG; <50 years of age), late-onset (LOMG; >50 years of age) and thymoma-associated MG (TAMG), all of which show thymic abnormalities [8]. Since the identification of the first and most abundant MG antibody target, the AChR, in 1976 [9,10], MG-associated Abs directed against other postsynaptic structures, such as muscle-specific kinase (MuSK) [11,12] and lipoprotein-receptor-related protein 4 (LRP4) [13,14] have been described. MuSK- and LRP4-associated MG patients typically depict no thymic abnormalities [1]. The presence of additional Abs against neuromuscular junction-associated molecules such as agrin [15], collagen Q (ColQ) [16] and voltage-gated potassium channel subfamily A member 4 (Kv1.4) [17] has been demonstrated in a few patients with MG. However, whether these Abs display any pathogenic function during disease remains undetermined. Aside from Abs against these extracellularly exposed soluble or transmembrane autoantigens, some MG patients harbour Abs against the intracellular proteins titin (present in 20–30% of AChR^+^ MG) [18], ryanodine receptor (RyR; present in 70% of TAMG) [19] and cortactin (present in 5–10% of AChR^+^ MG) [20]. Thus far, there is no evidence for an immediate pathogenicity of Abs directed against these intracellular targets in MG. While presence of anti-titin and -RyR is indicative of a more grave disease course [21], the pathomechanistic function of anti-cortactin remains largely obscure [20]. Seronegative MG most likely constitutes a heterogenous group of patients with low Ab titers, presence of low affinity Abs and Abs against clustered AChR or unidentified target structures [21,22].

**Figure 1:**
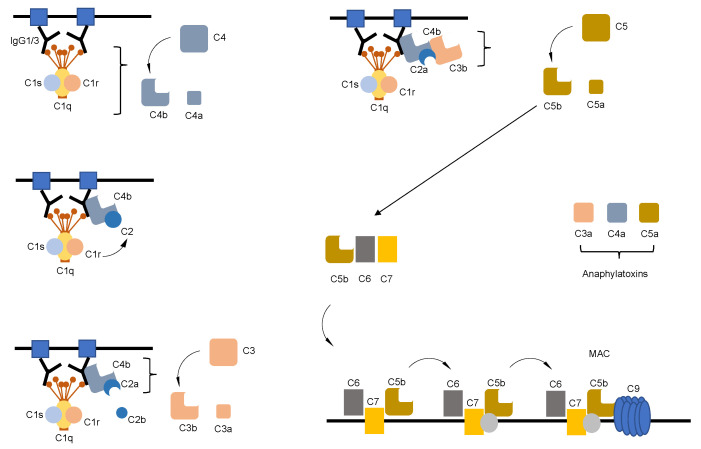
Classical complement pathway activation. Schematic illustration of the classical complement pathway. Antibody-mediated activation of C1 leads to formation of the C4bC2a complex, which cleaves C3 to produce C3b, which forms a complex with C4b and C2a. This complex cleaves C5 to produce C5a and C5b. C5b, initiates the lytic pathway and membrane attack complex (MAC) formation.

While IgM, IgG1 and IgG3 are highly competent in inducing the classical complement pathway, IgG2 shows only moderate capacity in doing so. IgG4, IgA, IgE and IgD are incompetent in classical complement pathway activation [23]. Abs in MG belong to the IgG class and a large body of evidence supports the induction of the classical complement pathway as the principal culprit of the postsynaptic membrane destruction in MG. The classical complement pathway is initiated via binding of C1q to the CH2 domain of IgG, which leads to the formation of the C1 complex consisting of C1q, C1r and C1s (C1qr^2^s^2^). The ensuing autoactivation of C1r is followed by activation of C1s and the subsequent activation of C4 and C2 leading to the assembly of the C3 convertase (C4bC2b). C4bC2b together with C3b yields the C5 convertase (C4b2b3b) which cleaves C5 into C5a and C5b. Finally, the terminal complement complex members C5b to C9 generate the membrane attack complex which is inserted in membranes to create cytotoxic pores and induce lysis of the target structure (Figure 1) [24].

It has been well established that both functional and morphological abnormalities of the thymus are associated with the development of some but not all MG entities. While thymic hyperplasia is common in EOMG patients, some patients with MG suffer from thymoma which classifies them as TAMG. Since thymic tissue of LOMG patients often appears atrophic and MuSK-/LRP4-associated MG patients are regularly found to have a normal organ, thymic involvement in the pathogenesis of these MG entities is considered unlikely [8]. MG forms associated with thymic pathologies (EOMG and TAMG) are believed to be triggered by an intrathymic mechanism, i.e., breakdown of central tolerance [21,25]. EOMG thymus is characterized by inflammatory hyperplasia and occurance of ectopic lymphoid follicles with germinal center-like structures, where antigen-activated B cells diversify their immunoglobulin genes by somatic hypermutation to generate high-affinity antibodies [25,26]. The functional impairment of Tregs in MG is likely to contribute to the intrathymic autoimmunization during the initiation of the disease [27].

Thymoma is diagnosed in 10–15% of MG patients [28]. Aside from TAMG, which constitutes the most frequently observed paraneoplastic autoimmune disease associated with this epithelial tumor, patients with thymoma are also more likely to develop systemic lupus erythematosus, thyreoiditis and rheumatoid arthritis [25]. Among different types of thymoma, those with greater thymopoietic propensity render patients more susceptible to developing TAMG [29]. In stark contrast to EOMG, patients with TAMG often develop in addition to anti-AChR also Abs directed against other muscle-associated proteins such as titin and RyR, which may reflect differential epitope expression of neoplastic epithelial cells compared to medullary thymic epithelial cells [30]. Furthermore, anti-AChR found in TAMG is not conformation-specific as observed in EOMG [30,31,32].

## 3. Autoantibody Specificities in MG

### 3.1. Anti-AChR

**Figure 2:**
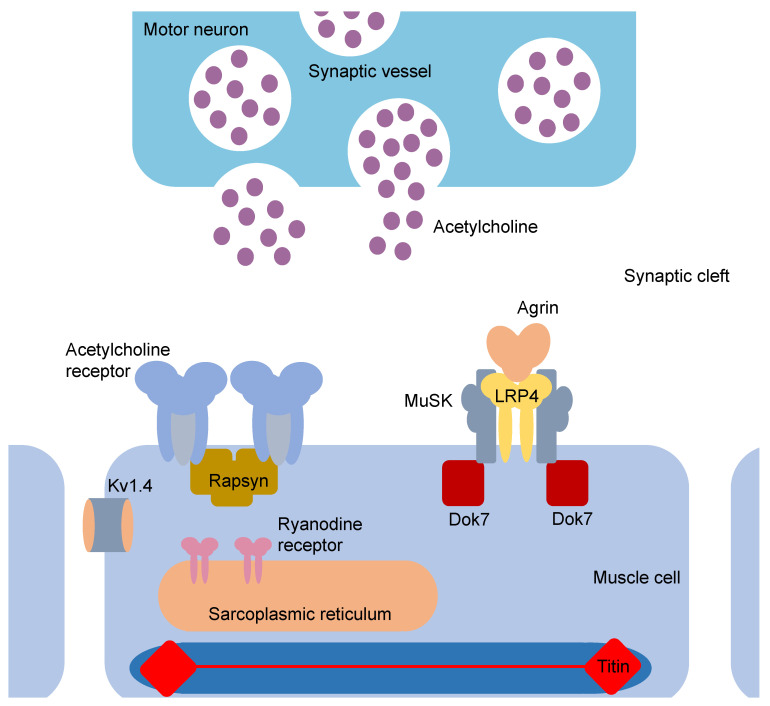
The neuromuscular junction in MG. Schematic illustration of the neuromuscular junction with targets for autoantibodies observed in patients with MG.

The most commonly detected disease-related Abs in MG (in around 80% of MG patients) are high affinity Abs directed against the muscle nicotinic AChR, a 250 kDa transmembrane glycoprotein composed of two α1-subunits, one β1-, one δ- and one γ-(embryonic/adult denervated muscle AChR) or ε-subunit (adult AChR), respectively [33] (Figure 2). AChR are members of the cys-loop ligand-gated ion channel superfamily and localized opposite to axon terminals at the end plate of the muscle post synaptic membrane. While depicting a certain degree of polyclonality, anti-AChR Abs are directed against the pentamer’s N-terminal extracellular domains, with the majority targeting conformational epitopes of the α1-subunits [34]. Passive transfer experiments into Lewis rats have shown that Abs directed against α1-subunit epitopes are of greater pathogenicity [35]. As anti-AChR-Abs most commonly belong to the complement fixing subclasses IgG1 and IgG3, they exert their pathogenicity in part via recruitment of the classical complement pathway triggered by the initial binding of C1 complex to the CH2 domain of IgG1/IgG3, ultimately leading to the generation of membrane attack complexes and the disintegration of the postsynaptic membrane [36,37]. Furthermore, through antigenic modulation, the binding of anti-AChR may lead to cross-linking of junctional AChRs which accelerates their internalization rate resulting in diminished receptor density on the postsynaptic membrane [38,39]. Competition with ligand binding sites as well as unphysiological conformational changes of the target structure represent additional mechanisms through which anti-AChR mediate pathogenicity [39,40].

### 3.2. Anti-MuSK

Abs against this muscle-restricted receptor tyrosine kinase are detected in 1–10% of patients with MG. The type I, single-pass transmembrane glycoprotein of 120 kDa is comprised of a large extracellular domain, a transmembrane domain, and an intracellular kinase domain. Following LRP4-dependent phosphorylation by binding of the motorneuronally derived ligand agrin, MuSK signals via rapsyn and Dok-7 to elicit clustering of AChR [40,41,42]. Anti-MuSK binds a structural epitope in the first Ig-like domain of MuSK, thereby preventing its interactions with LRP4 and ColQ, respectively, which ensues the inhibition of agrin-mediated MuSK phosphorylation [43]. This functional impairment of MuSK results in a reduced AChR density in the postsynaptic membrane and represents the molecular correlate of the clinical presentation in patients with MuSK-associated MG. Abs against MuSK belong by and large to the IgG4 subclass which renders them incompetent for complement activation. Due to their monovalency, they are unable to induce antigenic modulation [11,44].

### 3.3. Anti-LRP4

In total, 1–5% of all and around 19% of anti-AChR^neg^/-MuSK^neg^ MG patients show seropositivity for anti-LRP4 [13,14]. Recent studies report the occurrence of anti-LRP4 antibodies in MG patients with either anti-AChR or -MuSK antibodies and up to 23% of patients with amyotrophic lateral sclerosis have been found seropositive. LRP4 belongs to a family of structurally related, single-pass transmembrane proteins and amongst other sites is expressed in skeletal muscle where it is required during both pre- and postsynaptic differentiation processes. As a binding partner of agrin it facilitates activation of MuSK [42,45,46]. The majority of anti-LRP4 belong to the complement-fixing IgG1 subclass. It is possible that anti-LRP4 Abs damage postsynaptic structure via recruitment of complement proteins and impair signal transduction by diminishing MuSK function [47,48]. However, the precise mechanism through which these Abs mediate their pathogenicity during MG warrants further investigation.

### 3.4. Others

Further Abs against extracellularly exposed antigens detected in patients with MG include anti-agrin, anti-ColQ and anti-Kv1.4 [15,16,17]. Whether they exert direct pathogenic functions remains to be determined. In some patients with MG, Abs against intracellular antigens can be found: anti-titin, anti-RyR and anti-cortactin [18,19,20,49]. While presence of anti-titin and anti-RyR indicate a more severe clinical course, the degree to which these Abs directly mediate pathogenicity awaits further elucidation [12,50].

## 4. Clinical Spectrum of MG

Although the diagnosis is straightforward in most patients with typical symptoms and a positive Ab test, the phenotype of the disease and its clinical course including the response to immunotherapy is remarkably heterogeneous. For MG, different subtypes are described according to clinical phenotypes, thymic alterations, and age of disease onset or disease-associated Abs. With regard to clinical severity, the criteria of the *Myasthenia gravis* Foundation of America (MGFA) are commonly used [51]. The MGFA classification is designed to identify subgroups of patients with MG who share distinct clinical features or severity of disease that may indicate different prognoses or responses to therapy. One can distinguish between patients with exclusively ocular (MGFA I) and generalized symptoms (MGFA II-V). At 2 years after onset, 80–85% of patients with MG initially restricted to eye muscles progress to generalized MG [52]. Patients with a generalized presentation are further classified into a limb (II–IVa) or oropharyngeal (II-IVb) pronounced pattern of clinical involvement. Patients who have suffered a myasthenic crisis during the course of the disease are classified as MGFA V. Aside from the already mentioned clinical entities EOMG, LOMG and TAMG, one can differentiate juvenile MG and ocular MG. Around 10% of patients with MG are children under 17 years of age, which are categorized as juvenile MG. In this subgroup, patients with prepubertal onset (symptom onset before the age of 12) are distinguished from those with pubertal onset [53,54]. Generalization of symptoms occurs less often compared to adult MG patients. Moreover, the rate of spontaneous remissions is much higher than in adults [55,56,57]. Similar to the adult-onset MG, AChR Ab are mostly detected in both ocular and generalized MG [55,58]. Patients with juvenile MG seem to benefit from thymectomy without relevant signs of long-term immunodeficiency [59]. Ocular MG affects only the external eye muscles, including the levator palpebrae muscle, and manifests with ptosis and diplopia [60]. In general, ocular symptoms are often the initial manifestation of later generalized MG which usually occurs within the next 2 years [61,62,63]. In about 15–20% of all MG patients, manifestation remains purely ocular [64]. In contrast to adults and postpubertal children, ocular myasthenia is more common in prepubertal children and spontaneous remissions are possible [55,58,65]. Nearly 50% of the patients have Abs against AChR [33,61,63].

## 5. Current Immunotherapy of MG

The therapy of MG comprises symptomatic treatment, surgical resection for generalised AChR-Ab positive MG, and above all immunotherapeutic approaches. With regard to symptomatic treatment with anti-cholinesterases, only a small proportion of patients with generalised MG are clinically stable during the long-term course of the disease [66], leading to combination with immunosuppressive agents. Moreover, patients with MuSK-Ab positive MG often respond more poorly to anti-acetylcholinesterase treatment than patients with AChR-Ab positive MG, or already react to standard doses with side effects [67,68]. Corticosteroids are usually needed as a long-term therapy, whereby a slow dose increase at the beginning is required to prevent a short-term worsening of myasthenic symptoms. Once symptoms are stable or remitted, the high daily dose should be rapidly reduced with the aim of reaching the lowest dose that controls disease activity [69]. Immunosuppressive agents are essential for reducing corticosteroids to the lowest possible dosage and to prevent myasthenic crisis [5]. Initially and depending on the clinical severity, a stepwise regimen of anti-acetylcholinesterase, corticosteroids and azathioprine is used in generalised MG patients. Azathioprine is an antipurine antimetabolite that showed beneficial effects in MG patients [70]. Due to the delayed onset of action and the sometimes severe side effects during long-term application, alternative immunosuppressants are commonly used, however with no evidence of superior efficacy or safety profile [5,71]. In terms of myasthenic exacerbation or crisis, therapeutic plasma exchange or IVIg are promising options, however with only short term benefit [5,72,73].

The era of Ab therapies achieved stabilisation of MG, especially in patients with persistent disease activity on immunosuppressants. The two most well studied Abs in MG are eculizumab (see clinical studies) and rituximab [5]. For rituximab, a monoclonal Ab against the CD20 antigen, previous studies provide a promising option in the management of MG, particularly in patients with MuSK-Ab positive MG or patients with refractory disease [74]. Rituximab is not approved for MG [74], which limits its usage in daily clinical routine.

## 6. Fc-Receptor Biology in the Pathogenesis of MG

**Figure 3:**
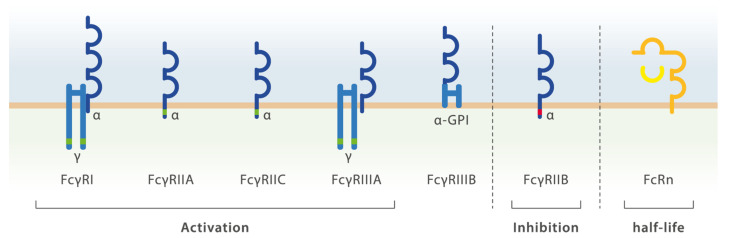
Human Fc receptors. Schematic illustration of human receptors with their respective signaling subunits (immunoreceptor tyrosine-based activation motif (ITAM) is highlighted in green, immunoreceptor tyrosine-based inhibition motif (ITIM) is highlighted in red). The FcRn is a non-classical Fc receptor which does not contain a signaling domain and regulates IgG half-life.

Receptors recognizing the Fc part of Igs provide an effective link between the innate and the antigen-specific adaptive branch of the immune system and can be divided according to the Ab class that they recognize (IgG, IgM, IgA, IgE, IgD). Upon binding of an Ab to a cognate epitope via its Fab domain, the Ig Fc part is available for Fc receptor binding [75]. In humans there are 5 Fcγ receptors recognizing IgG (FcγRI [CD64], FcγRIIA [CD32a], FcγRIIB [CD32b], FcγRIIIA [CD16a], FcγRIIIB [CD16b]) and the neonatal Fc receptor (FcRn), a structurally unrelated, MHC class I-like molecule with distinct functions (Figure 3).

Key functions of FcγRs include the facilitation of opsonized target phagocytosis and complement activation. FcγRs can be divided into activating (CD64, CD32a, CD16a/b) and inhibitory (CD32b) FcγRs. Aside from the high affinity FcγRI/CD64, all other FcγRs depict relatively low affinity for binding monomeric ligands. Thus, only multivalent ligation, such as in the case of immune complexes and opsonized particles trigger relevant signalling through these receptors. Complement-dependent cytotoxicity following IgG-Fc interaction is currently believed to be the predominant Fc-mediated pathomechanism during MG [6,76].

Aside from determining an Ab’s class and subclass, therefore matching up with a specific set of Fc receptors, the Ig Fc part also specifies the ability to initiate complement activation and fixation. Depending on their class and subclass, Abs largely differ in their propensity to activate the complement system. Generally, Ab-mediated activation of the complement system mainly refers to the classical complement pathway (Figure 1), however Abs are implicated also in the regulation of the alternative and lectin pathway of the complement cascade [24].

## 7. Targeting Fc Receptor Functions in MG

Novel molecules that may potentially target Fc receptors for therapeutical purposes in MG include recombinant Fc (rFc) multimers, Fcγ receptor (FcγR)-targeting agents and FcRn-targeting therapeutics.

### 7.1. rFc Multimers

Recombinant Fc (rFc) multimers constitute a novel group of tri- or hexavalent molecules with several IgG Fc moieties that are engineered with augmented affinity for FcγR-binding. Mechanistically, these multimers, by blocking accessible Fc receptor binding sites, prevent Ab immune-complex-mediated FcγR activation [77]. In a murine model of MG, treatment with the recombinant polyvalent IgG2a Fc multimer M045/GL-2045 showed similar efficacy as compared to IVIg [78]. Additionally, certain rFc multimers may exert their anti-inflammatory properties through inhibition of the classical complement pathway via interception and fluid-phase activation of C1q [79]. This has led to the purposeful engineering of novel analogues with limited ability to interact with low/moderate affinity FcγRs, but high avidity for C1q, making them de facto complement inhibitors, specifically of the classical complement pathway [80]. The added value of such Fc-rooted complement inhibiting agents over established biologics such as monoclonal anti-complement component C5 antibodies eculizumab or ravulizumab, awaits further investigation. The promising trivalent Fc oligomer CSL730/Fc3Y with avid binding properties to FcγRs but without FcγR activation potential has proven to broadly dampen FcγR-mediated cellular activation without activating immune cells and showed therapeutical efficacy in preclinical animal models of Ab-mediated autoimmune diseases [81]. However, thus far, there is no preclinical data on the effectiveness of this oligomer in experimental MG.

### 7.2. FcγR-Targeting Agents

This group is comprised of monoclonal Abs directed against FcγR, recombinant soluble FcγR and preparations of IVIg or Fc fragments with targeted Fc-glycosylation. Monoclonal anti-FcγR Abs have been engineered to target immunoglobulin-binding domains in activating FcRs. The anti-human FcγRIIIa antibody 3G8 has shown encouraging clinical efficacy in patients with primary immune thrombocytopenia but also elicited severe adverse effects, likely due to Fc-mediated FcγRIIIA crosslinking via ITAM signaling and consecutive aberrant immune activation [82]. The subsequent engineering of modified 3G8 with deglycosylated Fc (GMA161) depicted similar clinical efficacy as compared to the original 3G8. However, despite its reduced interaction with FcγR due to deglycosylation, GMA161 persevered in inducing clinical toxicity [83,84]. To date, there are no available data of monoclonal human anti-FcγR Abs in preclinical models of or patients with MG.

Soluble FcγR, such as the recombinant human FcγRIIb Valziflocept, are thought to execute their therapeutical potential by neutralizing circulating pathogenic immunoglobulins before they can bind and signal through autochthonous FcγR. While no data is currently available on patients with MG, phase II trials in patients with systemic lupus erythematosus and immune thrombocytopenia yielded promising results [85].

IVIg is a purified blood product preparation containing IgG Abs from thousands of donors per lot and due to its immunomodulatory properties has been increasingly applied in the treatment of several autoimmune diseases over the last decades [72,73,86,87]. Alongside therapeutic plasma exchange and immunoadsorption, IVIg constitutes an effective treatment for patients with MG in acute exacerbations [88,89]. IgG Fc regions contain a complex architecture of sugar moieties, the composition of which is critical for determining the operability and degree of Fc-mediated interactions such as complement fixation and ligation of FcγR. These Fc-associated carbohydrates are covalently linked to Asn297 of the CH2 domain with a *N*-acetylglucosamine (GlcNAc) core followed by terminal and branching residues including galactose, sialic acid, fucose, and GlcNAc. Post-translational modification of the Fc carbohydrate composition can be harnessed for therapeutical purposes [90]. IVIg preparations highly enriched for glycovariants with sialylated Fc core polysaccharides acquires remarkable anti-inflammatory properties [91]. Fucosylation of IgG Fc can hamper the binding to FcγRIII and reduce antibody-dependent cellular cytotoxicity [92]. Conversely, defucosylation of IgG Fc results in maximized efficacy of therapeutic depleting antibodies [93,94]. Furthermore, while IgG Fc sialylation compromises their efficacy to induce complement-mediated cytotoxicity (CDC), galactosylation of IgG Fc leads to CDC enhancement [95,96]. However, IgG Fc galactosylation has also been reported to facilitate anti-inflammatory effects via cooperative FcγRIIb/dectin-1-mediated suppression of the C5a receptor [97], exemplifying the degree of intricacy these glycoengineering approaches are confronted with in calibrating desired immunomodulatory effects.

### 7.3. FcRn-Targeting Therapeutics

**Figure 4:**
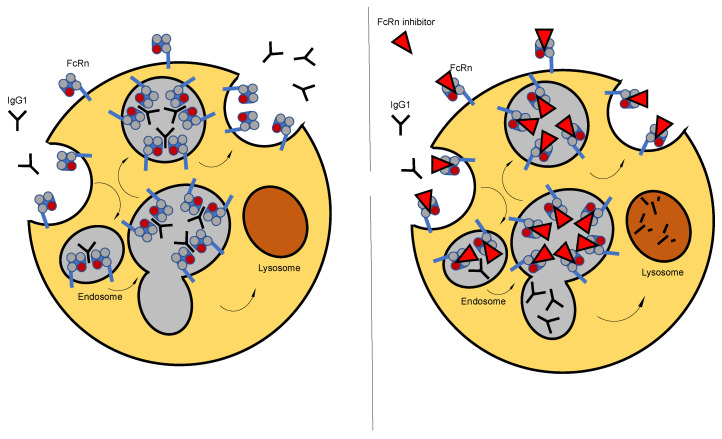
Mechanism of FcRn targeting. Left: Physiological FcRn mode of action. Right: FcRn inhibitor-mediated disruption of IgG recycling. The neonatal Fc receptor (FcRn), ubiquitously expressed in endothelial cells and in myeloid cells of the immune system, interacts with IgG and facilitates its internalization and subsequent recycling via a pH-dependent mechanism. FcRn does not interact with IgG at a neutral pH of 7.4. The acidic environment of the endosome following pinocytosis allows for efficient binding of IgG to the receptor, which prevents endosomal transport of IgG to the lysosome for subsequent degradation. FcRn-bound IgG is eventually recycled back to the cell surface and exocytosed. Exposure to a neutral pH facilitates release of IgG from the receptor. This FcRn-mediated recycling mechanism, ultimately leads to the half-life extension of all IgG subclasses and can be blocked by specific molecules that interfere with FcRn binding.

The FcRn, is a MHC class I like heterodimer consisting of a tripartite heavy α-chain associated with β-2-microglobulin and is amongst other tissues expressed in the vascular endothelium [98,99]. FcRn interacts with IgG (IgG:FcRn—2:1) and albumin (albumin:FcRn—1:1) via differential binding domains and facilitates their internalization and subsequent recycling. The receptor and its function were initially identified as part of a mechanism for intrapartum transfer of IgG across the placenta from mother to fetus in order to provide immune protection to the newborn [98,100,101]. As a transporter for IgG and albumin, FcRn regulates their serum homeostasis via a pH-dependent cellular recycling mechanism. While FcRn does not interact with IgG at a neutral pH of 7.4, the acidic environment of the endosome following pinocytosis of IgG allows for efficient binding of IgG to the receptor, which prevents endosomal transport of IgG to the lysosome for subsequent degradation. FcRn-bound IgG is eventually recycled back to the cell surface and exocytosed. Exposure to a neutral pH of 7.4 facilitates release of IgG from the receptor. This FcRn-mediated recycling mechanism, ultimately leads to the half-life extension of all IgG subclasses [102] (Figure 4).

With MG being an autoimmune condition, during which pathogenicity is largely exerted via detrimental Abs, removal and lowering of Igs have a long-standing place in the therapeutic armory of the disease. Therapeutic plasma exchange is commonly applied in patients with MG to physically remove pathogenic Abs as well as non-pathogenic Igs and other utile molecules. Although immunoadsorption is more specific, in that it may only remove Igs, non-pathogenic Abs are eliminated as well. Aside from therapeutic plasma exchange and immunoadsorption, IVIg are commonly used in treating MG exacerbations and crisis. Part of the proposed mechanism through which IVIg mediates their beneficial effects is thought to be shortening the half-life of pathogenic Abs via competitive binding of the infused IgG to FcRn [72,73]. However, besides acting as “bait” IgG, IVIg may execute their therapeutic efficacy through a range of mechanisms and their pleiotropic modulatory activity is prone to inducing adverse effects. Thus, the novel treatment approach of specifically targeting the FcRn via engineered high affinity Abs termed Abdegs (antibodies that enhance IgG degradation), thereby accelerating IgG catabolism, may lead to efficient, more tailored therapeutic options with less adverse effects [103]. The monoclonal anti-FcRn Ab 1G3 has proven to ameliorate the disease course in passive and active preclinical animal models of MG in a dose-dependent manner via effectively reducing serum levels of pathogenic Abs [104].

## 8. New FcR-Targeting Therapies: Evidence from Clinical Trials

### 8.1. Fc- and FcR-Targeted Therapies in MG

Given their relevance in numerous autoimmune diseases, classical FcRs have been considered as attractive therapeutic targets for the last three decades. However, a limited number of investigational drug candidates have been developed targeting FcγRs and most of them have been tested in patients with immune thrombocytopenia [105,106]. In MG, primarily FcRn-targeting treatment approaches have been developed and are currently being evaluated in several clinical trials (Table 1). FcRn-targeting therapies are designed to specifically and selectively target FcRn to increase the catabolism of (auto-)Abs. Therefore, a variety of approaches were used including monoclonal Abs against FcRn as well as binding Fc fragments [106]. Both therapeutic approaches lead to a significant decrease in serum IgG concentrations over time and correspondingly in MG associated Ab titres, which is reversible after treatment discontinuation [107,108,109,110,111]. In terms of infection risk, those drugs appear to target only IgG recycling and not affect other components of the innate and adaptive immune system [106,108]. However, long-term data on the relationship between sustained IgG reduction and risk of infection are still lacking. In light of the growing role of vaccination in times of the current coronavirus pandemic, further data on vaccination response in FcR-targeted therapies are urgently needed. Interestingly, the upcoming generation of monoclonal Abs are developed with respect to the recycling pathway by FcRn, leading to beneficial effects such as an extended dosing interval.

#### 8.1.1. Nipocalimab

Nipocalimab (M281) is a fully human IgG1 anti-FcRn Ab that reduces IgG levels in the blood [106,110]. In a phase 2, randomized, double-blind, placebo-controlled study, 60 patients with generalized AChR- or MuSK Ab-associated MG were enrolled in 4 different active and 1 placebo treatment arms (ClinicalTrials.gov Identifier: NCT03772587). Each participant received a total of 5 study IV infusions administered every 2 weeks. The primary endpoints were safety and the change from baseline in the MG-ADL score at day 57. Although official data were not present so far, an official press release announced that 52% of patients who received nipocalimab had a significant and durable reduction in MG-ADL scores for at least four consecutive weeks across all dosing regimens, compared to 15% of the placebo arm. Nipocalimab was well-tolerated and severe or serious treatment-related adverse events were not reported.

#### 8.1.2. Rozanolixizumab

Rozanolixizumab is a humanized, high-affinity, human IgG4 monoclonal Ab which targets FcRn [111]. Rozanolixizumab is administered subcutaneously and was investigated in a phase 2, randomized, double-blind, placebo-controlled, clinical trial in patients with AChR- and MuSK Ab positive generalized MG [112]. Key inclusion criteria were the clinical indication for IVIg or therapeutic plasma exchange as judged by the investigator and a Quantitative *Myasthenia gravis* (QMG) score of at least 11 points. In total, 43 patients were randomized to three once per week subcutaneous infusions of placebo or 7 mg/kg rozanolixizumab. Study participants were followed for 4 weeks after the last infusion and then were re-randomized to 3 doses of either 4 or 7 mg/kg rozanolixizumab. Primary endpoint was change from baseline to day 29 in QMG score. Beneficial changes from baseline to day 29 in QMG score for rozanolixizumab compared with placebo were not statistically significant, but the continuation of rozanolixizumab 7 mg/kg treatment in the second treatment phase led to further clinical improvements. Treatment with rozanolixizumab was safe and well-tolerated. The most common adverse event was headache. Of note, only one patient with MuSK Abs was included [112]. A phase 3, randomized, double-blind, placebo-controlled, multi-center clinical trial of rozanolixizumab is currently ongoing. The primary endpoint is the MG-ADL score change from baseline at day 43 among AChR Ab positive MG patients.

#### 8.1.3. RVT-1401

RVT-1401 is a human recombinant anti-FcRn monoclonal IgG1 Ab, which was developed for IV or subcutaneous administration [106]. The ongoing phase 2 clinical trial will evaluate the safety and pharmacodynamic effects of subcutaneous RVT-1401 in AChR Ab positive MG patients (ClinicalTrials.gov Identifier: NCT03863080). RVT-1401 is administered weekly for 6 weeks with an optional open-label extension phase.

#### 8.1.4. Efgartigimod

Efgartigimod is a human IgG1-derived Fc fragment which binds on Fc/FcRn [106,109]. In a phase 2 trial, 24 AChR Ab positive MG patients were randomized 1:1 to placebo or 10 mg/kg efgartigimod [107]. The IV infusions administered on days 1, 8, 15, and 22 with an 8-week observation period after the last infusion. The primary endpoint was safety and secondary endpoints included change from baseline in validated MG clinical outcome measures. No safety concerns were reported and the QMG and MG-ADL changes from baseline were statistically significant at day 8 (QMG) and days 29 and 36 (MG-ADL) [107]. Against the background of these promising results, a phase 3 randomized, double-blind, placebo-controlled, multi-center trial was initiated. First results have already been released reporting that the primary endpoint assessing the percentage of MG-ADL changes at 8 weeks in the AChR-Ab positive MG patients was met.

### 8.2. Differential Affinity of Therapeutical Complement Inhibitors to FcRn

Eculizumab is the first approved monoclonal Ab for patients with MG [5]. It is a humanized monoclonal IgG Ab that binds to the terminal C5 protein of the complement system and is designed to prevent the formation of the C5b-induced membrane attack complex which damage the neuromuscular junction. A randomized and placebo-controlled phase 3 trial included 125 patients with treatment refractory AChR Ab associated MG, defined as two already used immunosuppressive therapies with persistent relevant clinical symptoms. After an induction dose of 900 mg/week for four doses and a maintenance dose of 1200 mg/every two weeks, and an assessment period of 26 weeks was initiated. Although statistically significant findings were reported only for the secondary endpoints and not in the pre-defined primary endpoint of a change in total scores on the MG-ADL [4], long-term results of the extension trial documented rapid and sustained clinical improvement leading to minimal manifestation or pharmacological remission in more than 50% of MG patients [113]. Eculizumab undergoes continual nonspecific pinocytosis by endothelial cells and trafficking to endosomes with or without bound C5 factor for lysosomal degradation or recycling pathway via FcRn [114,115]. However, a significant amount of eculizumab is degraded in the lysosome rather than recycled back via the FcRn pathway. The resulting low amount of free eculizumab in the blood requires timely re-administration, which is essential for the neutralization of newly synthesized C5 [114,116]. Ravulizumab, a second generation anti-C5 monoclonal Ab has recently demonstrated similar efficacy and safety in patients with paroxysmal nocturnal hemoglobinuria [117]. Compared with eculizumab, ravulizumab is recycled more effectively through the FcRn pathway due to its increased affinity for the FcRn and rapid endosomal dissociation of the ravulizumab-C5 complex, allowing lysosomal degradation of C5 and a higher recycling rate of ravulizumab [114]. This results in an extended dosing interval of 8 weeks compared to 2 weeks for eculizumab. Consequently, a phase 3, randomized, double-blind, placebo-controlled, multi-center study was initiated to evaluate the safety and efficacy of ravulizumab in AChR Ab positive MG patients (ClinicalTrials.gov Identifier: NCT03920293).

## 9. Conclusions and Outlook

Therapeutic strategies that exploit FcRn function to enhance degradation of endogeneous IgG or increase serum half-lifes of therapeutic Abs are currently being developed and have the potential to substantially improve the management of MG. Compared to FcRn-targeting therapies, therapeutic modalities that modulate function or signaling of classical FcγRs are less far developed for MG and further research is needed to understand their biology and potential pathogenetic role in MG. However, given the relevance and therapeutic potential in several Ab-mediated autoimmune diseases, FcγR modulation could have a therapeutic merit in MG.

Better definitions of clinical outcomes and the identification of prognostic biomarkers will provide a rationale on how to position and fully exploit the clinical benefits of these new treatment modalities. Increased availability of FcR-targeting therapies and other biologicals could overcome limitations of current non-specific immunosuppressive therapies and might allow for the development of more targeted, personalized treatment algorithms to maximize treatment efficacy and safety.

## Figures and Tables

**Table 1:** Next-generation biologics in MG therapy.

Compound	Target	Neurologic Indications	Clinical Trials
**Nipocalimab**	FcRn	MG	Phase 2, NCT03772587 open-label extension trial, NCT03896295
**Rozanolixizumab**	FcRn	MG MG CIDP	Phase 2, NCT03052751 Phase 3, NCT03971422 Phase2, NCT03861481
**RVT-1401**	FcRn	MG	Phase 2, NCT03863080
**Efgartigimod**	FcRN	MG MG CIDP	Phase 2, NCT02965573 Phase 3, NCT03669588 Phase 2, NCT04281472
**Ravulizumab**	FcRn Complement factor 5	MG NMOSD	Phase 3, NCT03920293 Phase 3, NCT04201262

Ab, antibody; CIDP, chronic inflammatory demyelinating polyneuropathy; FcRn, neonatal Fc receptor; mAb, monoclonal Ab; MG, myasthenia gravis; NMOSD, neuromyelitis optica spectrum disorder.

## Abbreviations

Acetylcholine receptor	AChR
Antibodies that enhance IgG degradation	Abdegs
Antibodies	Abs
Collagen Q	ColQ
Complement-mediated cytotoxicity	CDC
Early onset MG	EOMG
Fcγ receptors	FcγR
IgG crystallizable fragment	Fc
Immunoglobulin G	IgG
Intravenous immunoglobulins	IVIg
Late-onset MG	LOMG
Lipoprotein-receptor-related protein 4	LRP4
Major histocompatibility complex	MHC
Muscle-specific kinase	MuSK
Myasthenia Gravis Foundation of America	MGFA
Myasthenia gravis	MG
Neonatal Fc receptor	FcRn
Quantitative Myasthenia Gravis score	QMG score
Regulatory T cell	Tregs
Ryanodine receptor	RyR
Thymoma-associated MG	TAMG
Voltage-gated potassium channel subfamily A member 4	Kv1.4
